# Do Young Children Understand Relative Value Comparisons?

**DOI:** 10.1371/journal.pone.0122215

**Published:** 2015-04-15

**Authors:** Joyce F. Benenson, Henry Markovits, Bjorn Whitmore, Christophe Van, Sara Margolius, Richard W. Wrangham

**Affiliations:** 1 Department of Human Evolutionary Biology, Harvard University, Cambridge, Massachusetts, United States of America; 2 Department of Psychology, Emmanuel College, Boston, Massachusetts, United States of America; 3 Département de psychologie, Université du Québec à Montréal, Montreal, Canada; Utrecht University, NETHERLANDS

## Abstract

Many forms of judgments, such as those used in economic games or measures of social comparison, require understanding relative value, as well as the more complex ability to make comparisons between relative values. To examine whether young children can accurately compare relative values, we presented children 4 to 7 years with simple judgments of relative value in two scenarios. Children then were asked to compare the relative values in the two scenarios. Results show that even the youngest children downgraded evaluations of a reward when another has a larger amount, indicating the ability to make relative value judgments. When asked to compare relative values however, only the oldest children were able to make these comparisons consistently. We then extended this analysis to economic game performance. Specifically, previous results using economic games suggest that younger children are more generous than older ones. We replicate this result, and then show that a simple change in procedure, based on the initial study, is sufficient to change young children’s choices. Our results strongly suggest that conclusions regarding young children’s pro-social motives based on relative value comparisons should be viewed cautiously.

## Introduction

One important difficulty when interpreting young children’s choices in tasks designed to detect basic motivational processes are possible effects of limited cognitive capacity. In particular, we argue here that decisions that require comparing relative values are more complex than they appear on the surface. For example, over the past decade, economic games used to study strategic interactions [1] and pro-social behavior [2–4] in adults increasingly have been used with young children. Such games often require participants to make decisions about distributing to or receiving rewards from other individuals. In these cases, decisions depend on both computation of relative values and comparison of relative values of differing choices. Relative value constitutes an important parameter in determining subjective evaluation of gains. Even non-human animals may base decisions on relative value [5]. Adults consistently modulate the subjective value of a given reward by considering how much is given to others in the immediate environment [6] For example, the subjective value of 5 units of reward increases when another individual has only 2 units, while it decreases when the other has 8 units. By 7 to 8 years of age, children consider that the subjective value of a reward decreases when others have more [7]. Additional evidence indirectly suggests that even younger children can make this relative value judgment. Young children aged 3–7 years do not like to receive less than another in both zero-sum and non-zero sum games [8–11]. Such results suggest that young children can make relative value judgments that discount the value of a reward when another has more. To be more specific, such judgments require that children be able to consider both the absolute value of an object (or the absolute level of a given performance) and the context in which the object is placed in order to make a single relative value judgment.

Critically many decisions, including those used in several forms of economic games, require *comparisons* of relative values. For example, one method often used in such games with children [4] involves giving the participant a choice between pairs of rewards, e.g. 2 for themselves and 0 for a partner, or 1 for themselves and 1 for a partner (this is the CSG option). A full comparison of these two choice sets requires being able to compare the relative value of 2 rewards (when the other has 0) to the relative value of 1 reward (when the other has 1). This constitutes a cognitively challenging task, since each relative value is itself a comparative calculation. In fact, comparing relative values requires an understanding akin to that involved in proportionality. Other forms of judgments also require some form of relative value comparisons, for example, using social information to make self-evaluations that involve placing an individual performance in ranked order with others, e.g.[12]. There is however no evidence that young children can make such comparisons (for reviews, [13, 14]).

Consistent with this analysis, there is in fact clear evidence that cognitive constraints can strongly influence young children’s judgments. Hook and Cook [15] examined age-related patterns of resource allocation as a function of completed work. Such allocation decisions theoretically require understanding proportionality, in the same way as relative value comparisons. Children’s allocation decisions in fact were directly related to their understanding of proportionality, which is a late developing ability. Consequently, Hook and Cook [15] concluded that younger children’s decisions might be affected by cognitive constraints as much as by other variables. Similarly, Damon [16] compared children’s conceptions of positive justice and their performance on a Piagetian assessment of logico-mathematical performance. He found a strong association between the two, rendering it impossible to disentangle cognitive ability and conceptions of positive justice.

These studies suggest that cognitive limits might prevent children younger than 7 years of age from making judgments that are consistent with their real motivations when these judgments require comparisons of relative value. Despite this, there has been little effort to examine how children’s understanding of these tasks might impact on their judgments. Accurate interpretation of young children’s decisions both in economic games and in other forms of judgments requiring relative value comparisons necessitate determining at what age children are able to consistently make relative value comparisons.

The following studies consequently were designed to investigate two hypotheses: 1) that young children have difficulty in making relative value comparisons even when these are made as explicitly as possible and 2) that very young children’s judgments in some economic games is attributable to this cognitive difficulty.

## Ethics statement

All studies were approved by the IRB of Université du Québec à Montréal or Emmanuel College. Written consent was obtained from the school and the parents of each child examined in these studies.

## Study 1

In order to examine the question of whether young children can make consistent relative value comparisons, we constructed sets of paired scenarios. In these, the target character always received two rewards, while the yoked character’s number of rewards varied. Children first were asked to make simple relative value judgments by rating the happiness of the target separately in two scenarios. We then assessed children’s ability to compare relative values in the two scenarios. To reduce personal motivations and simplify evaluations, we depicted cartoon characters receiving gifts. Children ranged from 4 to 7 years.

## Method—Study 1

### Participants

A total of 122 children from Montreal, Canada participated in this study. Of these, 37 were in preschool, age 4 years (Age: M = 4.59 years, 18 boys, 19 girls), 29 were in kindergarten, age 5 years (Age: M = 5.73 years, 12 boys, 17 girls), 17 were in Grade 1, age 6 (Age: M = 6.58 years, 12 boys, 5 girls), and 39 were in Grade 2, age 7 years (Age: M = 7.83 years, 17 boys, 22 girls). All children were native French speakers and were of middle to lower-middle class.

### Procedure and material

All children heard the identical introduction which provided a brief description of a daycare center for cats. The *target* cat then was introduced. Following this, participants were told that at this day care, every day some cats were given presents. Children were told that they were going to be asked how happy the *target* cat was when receiving different numbers of presents using a 5-point scale using cartoon faces progressing from very unhappy to very happy (which had been pretested to ensure that children could use the scale). To verify that children understood the task, children first were asked to indicate how happy the *target* cat would be if the *target* cat received 0, 2, and 4 presents. This was to verify that children believed that having more presents made the *target* cat absolutely happier.

Then, in each scenario, three drawings on cardboard backing were displayed. The first drawing always showed the *target* cat in front of a desk on top of which were 2 colored boxes depicting the presents. Next to the target cat was an empty desk. The child was told that the *target* cat had been given 2 presents in the morning. The second drawing always showed the *classmate* cat behind the second desk, on top of which were X (ranging from 0 to 4) number of boxes. The child was told that the *classmate* cat had received X numbers of presents. Finally, the third drawing always showed the *target* cat and the presents on its desk, plus the classmate cat’s presents on its desk, but not the second cat. The *classmate* cat was not present in the third drawing so as to emphasize that the comparison was between the rewards, and that only the *target* cat’s happiness was to be evaluated. Participants were told that the *classmate* cat had left to go to the bathroom, but had left its presents on its desk. Participants then were asked to rate the happiness of the *target* cat, using the 5-point scale. Children’s judgments were noted by the experimenter. The procedure then was repeated for the second scenario, which differed only in the number of presents received by the *classmate* cat, and the time of day (morning or afternoon).

**Fig 1:**
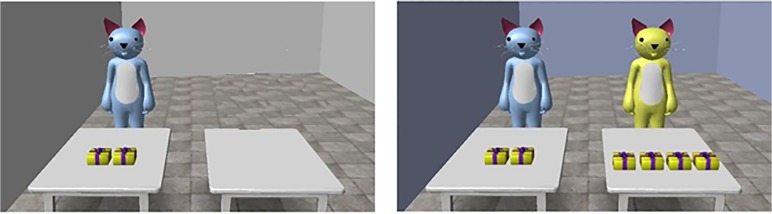
Example of one multiple comparison in which the child must evaluate in which of the two scenarios the *target* cat is happiest. On the left, the *classmate* cat receives no presents. On the right, the *classmate* cat receives 4 presents. The *target* cat always receives 2 presents. The child had previously rated the *target* cat’s happiness separately in each of these pictures.

After making these two separate relative value judgments, we asked children to compare the two situations. To make this comparison as simple as possible, we showed the child the two final drawings of each scenario side-by-side, both depicting the *target* cat and its presents and the *classmate* cat’s presents, but no *classmate* cat (see Fig 1 for an example). In both drawings, the *target* cat had two presents on its desk. In one drawing the *classmate* cat always had more presents than the *target* cat, while in the other drawing the *classmate* cat had the same number or fewer presents than the *target* cat. Participants then were asked whether the *target* cat was happier or equally happy in the morning (while pointing at the third drawing from scenario 1) versus the afternoon (while pointing at the third drawing from scenario 2). In all cases, children received no feedback after responses.

We created two pairs of scenarios with clear relative value comparisons. In each pair, in one scenario the *classmate* cat had more presents than the *target* cat, whereas in the other scenario, the *classmate* cat had fewer than the *target* cat (each scenario pair simply differed by the absolute difference). In all cases, the *target* cat received 2 presents. The first pair consisted of one scenario in which the *target* received 2 presents and the *classmate* received 1 present, and a second scenario in which the *target* received 2 presents and the *classmate* received 3 presents. The second pair consisted of the *classmate* receiving 0 presents in one scenario and 4 presents in the other. To simplify identification we refer to scenarios by the *classmate* cat’s number of presents only. For example, when one scenario showed the *classmate* cat with zero presents (*target* 2 versus *classmate* 0) and the paired scenario showed the *classmate* cat with four presents (*target* 2 versus *classmate* 4), this was identified as 0 versus 4. The order of presentation of the pairs was counterbalanced within each age group. In addition, within each pair, the presentation of each scenario on the left or right side was counterbalanced. Each participant thus made a total of 6 judgments in two sets of 3. The first set comprised 2 separate relative value judgments followed by a relative value comparison, followed by the second set, with 2 separate judgments and a comparison.

## Results and Discussion—Study 1

To ensure that children equated more presents with greater happiness, we first compared children’s happiness ratings for three values (0, 2 and 4 presents) using a repeated measures analysis of variance (ANOVA) with number of presents as the repeated factor and age as the independent variable. This yielded only a significant effect of number of presents, *F* (2, 112) = 16.67, *p* < .001. Post hoc comparisons using t-tests with Bonferroni corrections indicated that at all ages, mean happiness rating for 0 presents (*M* = 1.18, *SD* = 0.74) was less than for 2 presents (*M* = 3.08, *SD* = 1.26) which was less than for 4 presents (*M* = 4.73, *SD* = 0.52).

### Understanding of simple relative value

**Fig 2:**
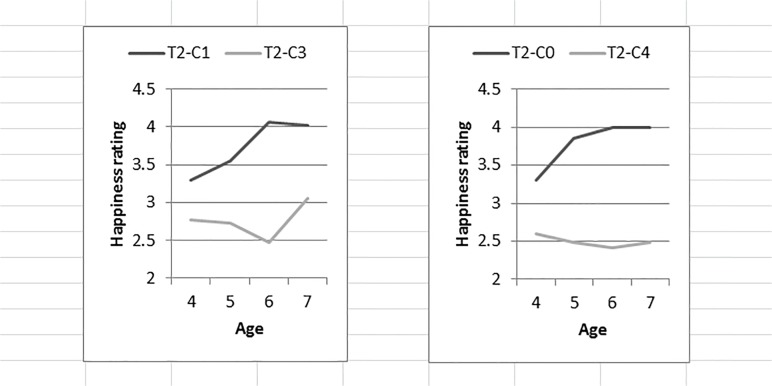
Performance on the Simple Relative Value Task. Mean ratings of the target cat’s happiness for each scenario pair by age with T = Target cat and C = Classmate cat.

We next compared children’s ratings of the *target* cat’s happiness in each of the two scenarios for all pairs (see Fig 2). Accordingly, we performed a repeated measures ANOVA with mean ratings of the target’s happiness in each of the two scenarios with number of presents of the *classmate* cat as the repeated factor and age and gender as independent variables. Follow-up Tukey’s tests with *p* < .05 were utilized where appropriate.

For Pair 1 (1 versus 3), only a significant main effect of the *classmate* cat’s presents, *F*(1, 114) = 32.77, *p* < .001, $\eta_{p}^{2}$ = .223 was observed. At all ages, children rated the *target* cat as significantly happier when the *classmate* cat received one present (*M* = 3.70, *SD* = 1.32) than when the *classmate* cat received three presents (*M* = 2.82, *SD* = 1.35).

Pair 2 (0 versus 4) showed exactly the same pattern as Pair 1. The ANOVA showed only a significant main effect of the *classmate* cat’s presents, *F* (1, 113) = 44.27, *p* < .001, $\eta_{p}^{2}$ = .281. At all ages, children rated the *target* cat as significantly happier when the *classmate* cat received no presents (*M* = 3.74, *SD* = 1.40) than when the *classmate* cat received four presents (*M* = 2.54, *SD* = 1.39).

In summary, these results demonstrate that children 4 years of age and older consistently evaluate the target cat as less happy when the classmate cat receives more presents relative to the target cat than when the classmate cat receives relatively less presents.

### Comparisons of relative values

Next, we examined children’s ability to make explicit comparisons of the target’s relative happiness. For every pair of scenarios, we showed the children the third picture in each paired scenario and asked whether the *target* cat would be happier in one scenario or the other or equally happy in both. Table 1 shows the percentage of children explicitly stating that the *target* cat was happier in one of the two scenarios. The third option, “equally happy”, is not shown but consists of 100 minus the sum of the two other options.

**Table 1:** Children’s Choices in the Relative Value Comparison Task.

	Scenario (1 versus 3)			Scenario (0 versus 4)		
Age	1	3	Equal	0	4	Equal
**4**	27%	32%	41%	24%	32%	44%
**5**	28%	21%	51%	21%	28%	51%
**6**	41%	12%	46%	41%*	0%	59%
**7**	54%**	10%	36%	56%**	10%	34%

Values indicate percentage of participants at each age level explicitly evaluating the target cat (who has two presents) as happier in one or the other scenario or equally happy in the explicit multiple comparison task.

*p < .05

**p < .01

For each comparison, we initially used a global chi-square analysis to calculate whether the overall distribution of choices differed from chance (.33). When this was the case, we used post-hoc chi-square analyses with a Bonferroni correction (p < .015) to examine whether one of the two scenarios was chosen more often. Critically, none of the response distributions differed from chance among 4- and 5-year olds. Among the 6-year old children, the *target* cat was evaluated as significantly happier when the *classmate* cat had fewer presents than the *target* cat for the (0 versus 4) pair, *X* ^2^(2) = 9.27, *p* < .01. The 7-year olds evaluated the *target* cat as significantly happier *when the classmate cat had fewer*, for both pairs: (0 versus 4), *X* ^2^(2) = 12.46, *p* < .01, (1 versus 3), *X* ^2^(2) = 11.21, *p* < .01.

These results show that 7-year olds’ comparisons were completely consistent with their simple relative value judgments in all cases. The 6-year olds show a similar pattern in the more extreme pair (0 versus 4). In contrast, the 4- and 5-years olds did not show any such tendency. The developmental pattern is thus consistent with the idea that the ability to make relative value comparisons that are consistent with individual relative value judgments develops over the age range examined here, with 4- and 5-year olds unable to do so, and 6-year olds beginning to exhibit this capacity.

The results of this study produce a clear developmental pattern. Children as young as 4 years of age generally make consistent simple relative value evaluations. Specifically, at all ages, ratings of the happiness of the target cat (who always had 2 presents) when the classmate cat had fewer than 2 presents were greater than happiness ratings when the classmate cat had more than 2 presents. Although earlier results suggest that young children are capable of making consistent relative value judgments, this provides direct evidence that children as young as 4 years of age can do so. In marked contrast, when children were directly asked to compare the same two scenarios used to make the simple relative value judgments, they found this very difficult. It was not before 7 years of age that children were able to do so in a way that was completely consistent with the individual evaluations. More detailed analyses indicate that both the older and the younger children were making comparisons on the same basis, but that the latter were doing so less efficiently, consistent with the idea that relative value comparisons have a greater cognitive load. Thus, these results are the first to show that while young children are able to make simple relative value judgments (in particular that they understand that having less than another decreases the value of a reward), the ability to compare two relative values, is more complex and develops later. This in turn suggests that when young children are asked to make such comparisons, they might do so by using some form of partial information. For example, they might simply consider only the rewards given to another. This could lead them to choose a situation where maximizing the reward given to another person might be the result of such a choice, even when this would be inconsistent with relative values judgments.

The distinction between making a simple relative value judgment and comparison of relative values can be used to understand seemingly divergent results in other forms of judgment. For example, research on self-evaluation shows that children as young as 4–5 years are able to modulate self-evaluations (a form of social comparison) appropriately when presented with one other child’s performance [17]. In contrast, children younger than 7–8 years are unable to utilize social comparison information to make accurate self-evaluations involving rank orders, e.g. [12]. Typically younger children overestimate their relative rank compared to others when asked to do so [13, 14]. However, if one considers that rank ordering evaluations requires *comparing* social comparison judgments, then the difficulty of the younger children can be attributed not to their lack of understanding of social comparison, but to the complexity of the comparisons required.

These results suggest that conclusions regarding young children’s judgments when these require relative value comparisons must be drawn cautiously, since their inability to make these comparisons could lead to judgments that do not reflect their basic motivations. In the following studies, we extend this analysis to examine one recently obtained set of results that appear to show that young children are more generous than older ones. Finally, it should be noted that the developmental differences found in this study must be considered to be a combination of age and schooling, factors which cannot be easily disentangled.

## Study 2

Past studies of adults in hunter-gatherer tribes to modern societies demonstrate that in the dictator game in which individuals are given multiple units of a valuable currency and asked whether they wish to donate anonymously to an unidentified community member, average donations vary between 25–47% of their currency units [18]. Given a clear tendency not to share equally, it is striking that in non-zero sum games across highly diverse cultures children younger than age 7 appear to behave more generously than older children and adults (i.e. they share 50% or more of their rewards [4, 11]. On the face of it, these reports about children younger than 7 appear to suggest that generosity may be even more basic to human behavior than self-interest. An alternative interpretation is suggested by the results of Study 1, i.e. is that younger children might not fully understand the task, since dictator games require relative value comparisons in order to fully comprehend the nature of the choices available to participants[4].

In Study 2, we first attempt to replicate results showing that younger children behave generously in a non-zero sum game. We constructed a modified version of the non-zero sum game used in previous studies. Children at two age levels (5–6 years of age and 7–8 years of age) were given two fake coins (exchangeable for toys) to keep for themselves. We then asked them to select an envelope to donate to a classmate, from among envelopes containing 0, 1, 2, 3, or 4 coins, in a single trial. We predicted that the younger children would be more generous than the older ones.

## Method—Study 2

### Participants

24 5–6-year-old kindergarten children and 24 7–8 year-old second grade children from low income multi-ethnic schools in Boston participated.

### Procedure and materials

Each child was interviewed individually outside the child’s classroom. We gave each participant two coins, across from which we placed an envelope containing two coins, flanked by two envelopes on one side that contained no coins and one coin, and two envelopes on the other side that contained three coins and four coins (with order of sides counterbalanced). Next, the child was asked to choose one of the envelopes to donate to another child in the class. The experimenter stressed that the donor and recipient would remain unknown to one another, then turned around while the child placed the envelope with the number of coins s/he wished to donate inside a party bag and the leftover envelopes in a garbage can.

## Results and Discussion—Study 2

**Fig 3:**
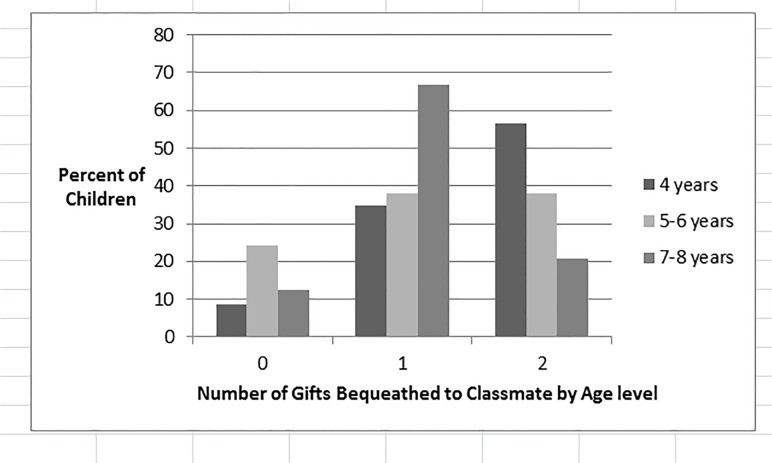
Children’s Donations by Age. Percentage of children within each age level giving each number of coins when child receives two coins and can donate 0–4 coins in Study **2**.

Results showed that, as predicted, more 5–6 year-old children than 7–8 year-old children donated four coins, *X* ^2^(1) = 4.75, *p* = .03 (see Fig 3). For the younger children, the modal choice was to donate four coins, which occurred significantly more often than would be expected by chance, *binomial test*: *p* = .003. In contrast, for the older children, the modal choice was to donate one coin, and this occurred more often than would be expected by chance, *binomial test*: *p* < .001. The results of this study replicate the finding that children under age 7 are more generous than older children [4, 11].

However, the results of Study 1 suggest that one possible explanation for young children’s apparent generosity is that they will use available cues to opt for a cognitive short-cut, when asked to make difficult relative value comparisons. For example, children might choose the most perceptually salient option [19] leading to preferential choice of the largest number of rewards.

## Study 3

To examine the hypothesis that children’s generosity did not reflect their motivations, we created a simplified choice procedure (referred to as the *simple relative value* task). In this, children had to make only one relative value decision at a time. In one of these, they were given 2 stickers for themselves with 4 stickers for an anonymous classmate. The child was asked if s/he was happy with how the stickers were distributed, and given the option to change the number given to the classmate. The simple relative value tasks were designed to make the comparison between the child’s own reward and the amount given to the classmate explicit and easily processed. In other words, this task explicitly reduces the cognitive load required by a full relative value comparison, by decomposing it into two simpler judgments which Study 1 shows that children have the ability to make consistently. This should allow the children to directly focus on the relative values, before attempting to compare them. Half of the children were administered a *multiple relative value* task exactly replicating Study 1. The other half were given two separate *simple relative value* tasks first; for each they received 2 stickers, but the classmate was given either 4 or 1, followed by the multiple relative value task.

Our first hypothesis is that more children would report being happy when the classmate was given 1 sticker (higher relative value for child) than when the classmate was given 4 stickers (lower relative value for child). If young children’s main motivation is indeed generosity, then more children should report being happy when 4 stickers were given to the classmate.

Our second hypothesis was that children who received the simple relative value tasks and then the multiple relative value comparison task would find it easier to focus on the relevant comparisons and consequently would be less generous on the latter.

## Method—Study 3

### Participants

70 5- to 6-year-old children from two middle-class preschools in Montreal, Canada participated in the study.

### Procedure and materials

The procedure for the multiple relative value task was identical to that of Study 2, except that stickers were provided rather than tokens. The two simple relative tasks began by placing 2 stickers directly in front of the child. Next, the child was told that inside a bag beside the experimenter were pieces of paper that indicated how many stickers an anonymous same-sex classmate would receive. Finally, the child was informed that if s/he was not happy with the number of stickers the classmate would receive, they should tell the experimenter who then would pick another number from the bag. At this point, the child was reminded that s/he had 2 stickers.

The experimenter then drew a paper from the bag, read the number written on it, and informed the child that the classmate would receive either 1 or 4 stickers. The child was asked if he/she was happy with this. If the child stated that s/he was not happy, then the experimenter drew another paper from the bag. This paper was read silently by the experimenter who placed an envelope with an invisible number of stickers at the place reserved for the classmate’s rewards. If the child asked how many stickers the classmate received, the experimenter simply said that the child would find out later. The same procedure then was repeated for the second simple relative value task, except that the number drawn from the bag differed. The order of the number of recipient stickers (1 or 4) was alternated. Half the children were given only the multiple relative value task. The other half were given the two simple relative value tasks followed by the multiple relative value task.

## Results and Discussion—Study 3

Initial analysis of the *simple relative value* tasks showed that the internal order in which the two tasks (4 or 1) were administered had no effect. We then examined the number of donor children who accepted or rejected the recipient’s number of stickers (see Table 2). Consistent with our hypothesis, the percentage of donors who indicated they were not happy and therefore requested that the experimenter change the reward (reject) when the classmate received 4 stickers (M = 55.9%) was significantly greater than the percentage who rejected the reward when the classmate received 1 sticker (M = 17.6%), McNemar’s test: *X* ^2^ (1) = 7.58, *p* < .01.

**Table 2:** Children’s Responses to the Simple Relative Value Judgments.

4 to classmate	1 to classmate	*n*
**Accept**	**Accept**	12
**Accept**	**Reject**	3
**Reject**	**Accept**	16
**Reject**	**Reject**	3

Number of children accepting or rejecting giving 4 or 1 to classmate.

**Fig 4:**
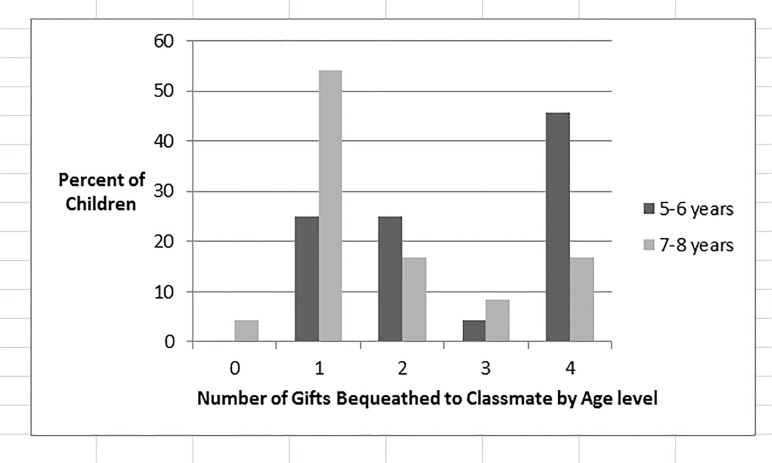
Children’s Choices on the Multiple Relative Value Condition by Order. Number of stickers given to classmate in the Multiple relative value condition as a function of Order (Multiple first, Simple first).

We next examined the number of children who donated 0, 1, 2, 3, or 4 stickers to the classmate when the multiple relative value task was administered first or after the simple relative value tasks (see Fig 4). Replicating Study 1, when only the multiple relative value task was administered, the modal response was to donate 4 stickers at a rate significantly greater than chance (.2), *binomial test*: *p* < .001. In contrast, children’s choices on the multiple relative value task differed when this task was given after the two simple relative value tasks, *X* ^*2*^(4) = 17.13, *p* < .01. In this case, the modal response was 1, which was chosen at more than chance level, *binomial test*: p < .01, like the 7–8 year old children in Study 1. In addition, the number of children choosing 4 stickers was not different from chance, *binomial test*: *p* = .17.

## General Discussion

The results of Study 1 clearly show that young children find it difficult to make comparisons of relative values that are consistent with their simple relative value judgments. While children as young as 4-years of age modulate the value of a reward to account for the reward possessed by another, in line with adults’ relative value judgments, they cannot compare relative values consistently before 6 or 7 years of age. This implies that young children’s performance on tasks that require such comparisons must be viewed with caution.

This has several potential implications. The most direct, which we have examined in Studies 2 and 3 concerns young children’s performance on economic games used to examine prosocial motivations. Typically, such games require children to donate rewards to another child when they have been given a reward themselves, or between pairs of rewards, e.g. 2 for themselves and 0 for a partner, or 1 for themselves and 1 for a partner. We have argued that such decisions require relative value comparisons, which are difficult for young children. Such a cognitive difficulty would lead to children making judgments based on only a single dimension, which would not adequately mirror their real motivations. We specifically re-examined some recent results that suggest that young children show a high degree of generosity in these games, which disappears in older children [4]. Such results, which have been also found using in other contexts [11] suggest that generosity might be a very primitive social motivation. We thus replicated this basic finding in Study 2, using a method designed to highlight the cognitive aspects of children’s decisions. The results of Study 3 also show that children younger than 7 years of age gave more to an anonymous same-sex classmate than they received themselves on tasks that required multiple comparisons. However, Study 3 shows that when the choices were simplified to highlight relative value with a procedure that explicitlycompared children’s own versus a classmate’s rewards one comparison at a time, children’s generosity was reduced. In fact, when children were given the simple comparison task first, their subsequent choices on the more standard multiple choice task closely resembled the behavior of children two years older. In other words, our results suggest that the complexity of the relative value comparisons demanded of young children is directly responsible for their apparent generosity in economic games. When task demands are simplified and the relevant comparisons made more explicit, this generosity disappears.

These findings demonstrate that young children’s choices in economic games and by extension any other form of judgment requiring relative value comparisons can be highly influenced by simple changes in procedure. It should be noted that a recent study [20] has found a similar variation in results when social behavior is examined in chimpanzees using methodologies that might create additional task complexity. Caution is therefore required in interpreting whether these choices reflect social motivations. Our results also suggest that young children (5–6 years old) did not exhibit generosity towards classmates. Rather, when given a procedure that was simple and easily understood, children preferred that classmates receive less than themselves, similarly to the behavior of older children. Future research must interpret with caution judgments designed to assess motivations of children under 7 years of age. In particular, it is vital to show that children understand the consequences of their choices.

## Supporting Information

S1 Data Set Responses to Simple and Muliple Relative Value Tasks in Study 1. (XLSB)

S2 Data Set Children’s Donations by Grade and Sex in Study 2. (XLSB)

S3 Data Set Children’s Donations and Responses to the Simple Relative Value Task by Order in Study 3. (XLSB)
