# Correction: Do Young Children Understand Relative Value Comparisons?

**DOI:** 10.1371/journal.pone.0128732

**Published:** 2015-05-15

**Authors:** Joyce F. Benenson, Henry Markovits, Bjorn Whitmore, Christophe Van, Sara Margolius, Richard W. Wrangham

There is an error in Fig 3, “Children’s Donations by Age”. Please see the corrected Fig 3 and its legend here.

**Fig 3:**
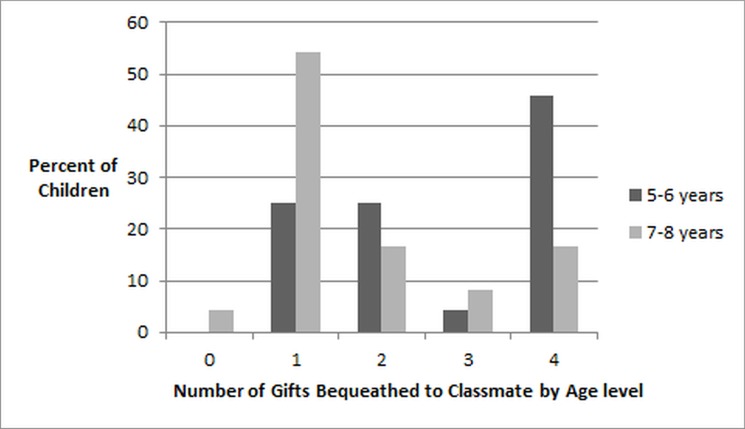
Children’s Donations by Age. Percentage of children within each age level giving each number of coins when child receives two coins and can donate 0–4 coins in Study 2.

There is an error in Fig 4, “Children’s Choices on the Multiple Relative Value Condition by Order”. Please see the corrected Fig 4 and its legend here.

**Fig 4:**
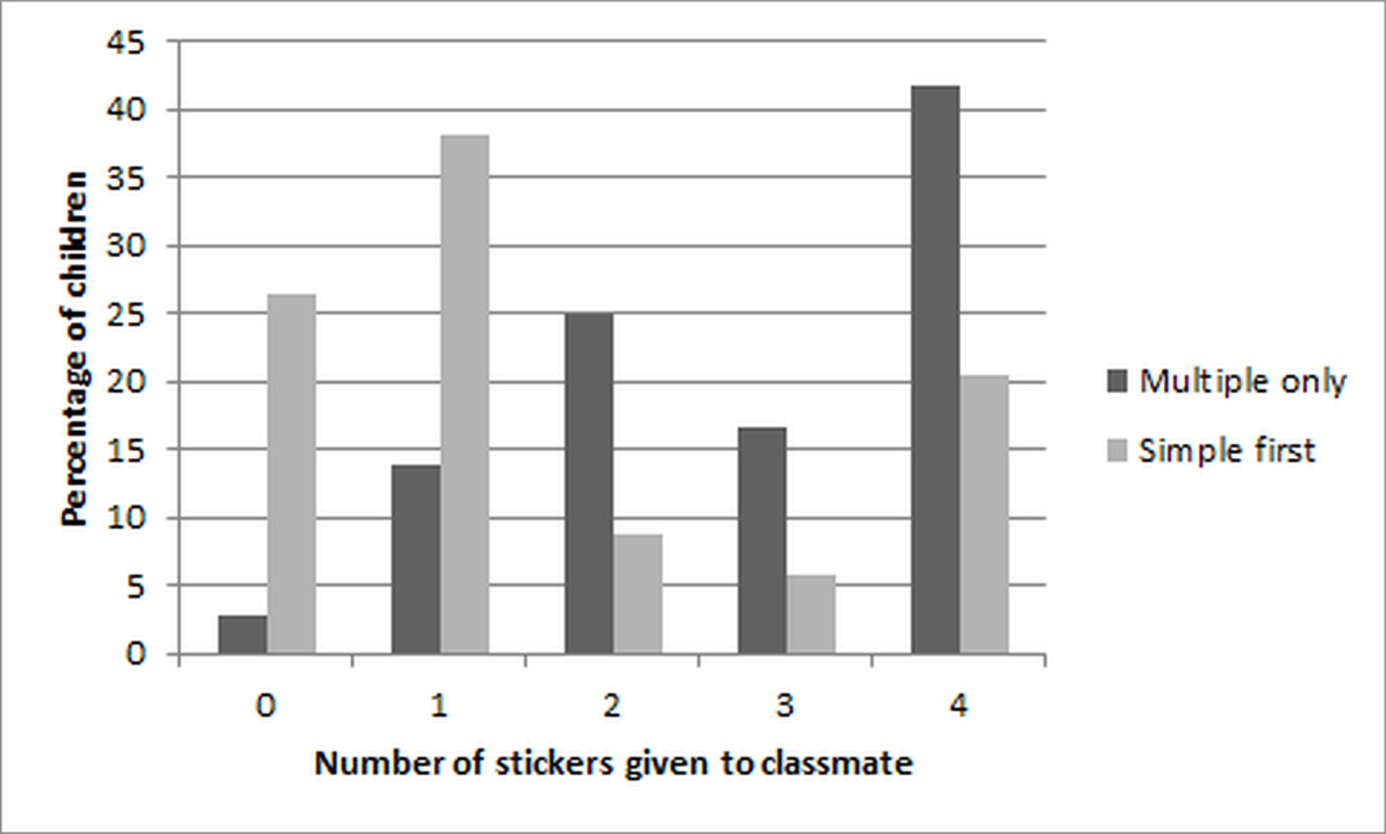
Children’s Choices on the Multiple Relative Value Condition by Order. Number of stickers given to classmate in the Multiple relative value condition as a function of Order (Multiple first, Simple first).
